# Supplementary material for: Monomeric prefusion structure of an extremophile gamete fusogen and stepwise formation of the postfusion trimeric state
Source: Nat Commun. 2022 Jul 13;13:4064. doi: 10.1038/s41467-022-31744-z (PMC9279424; doi:10.1038/s41467-022-31744-z)
Supplement: Supplementary file 1 — Supplementary Information [file 41467_2022_31744_MOESM1_ESM.pdf]

Supplementary Information for

Monomeric prefusion structure of an extremophile gamete fusogen and stepwise formation of the postfusion trimeric state

Juan Feng [1,2], Xianchi Dong[1,2,3], Yang Su[1,2,4], Chafen Lu[1,2] and Timothy A Springer[1,2]*

[1]Program in Cellular and Molecular Medicine, Department of Pediatrics, Boston Children's Hospital, Boston, MA, USA

[2]Department of Biological Chemistry and Molecular Pharmacology and Harvard Medical School, Boston, MA, USA

[3]Current Address: School of Life Sciences, Nanjing University, Nanjing, China

[4]Current Address: Department of Cell Biology, Harvard Medical School, Boston, MA, USA

*Correspondence to: 3 Blackfan Circle, Rm. 03-103, Boston, MA 02115, 617-713-8200, springer@crystal.harvard.edu

This PDF file includes:

Supplementary Figure 1. D2.1 and D2.2 re-orientation between pre- and postfusion states of HAP2 and Class II fusogens.

Supplementary Figure 2. Negative stain EM class averages of Cyani HAP2 ectodomain.

Supplementary Figure 3. Trimerization of Cyani HAP2 at pH 5.0 at different time points in β-DDM.

Supplementary Figure 4. EM class averages of Chlamy HAP2 at pH 7.5 with or without β-DDM at 23°C.

Supplementary Figure 5. Negative stain EM class averages of Cyani HAP2 D1D2.

Supplementary Figure 6. Reliablity of AlphaFold predictions.

Supplementary Table 1. Statistics of X-ray diffraction and structure refinement.

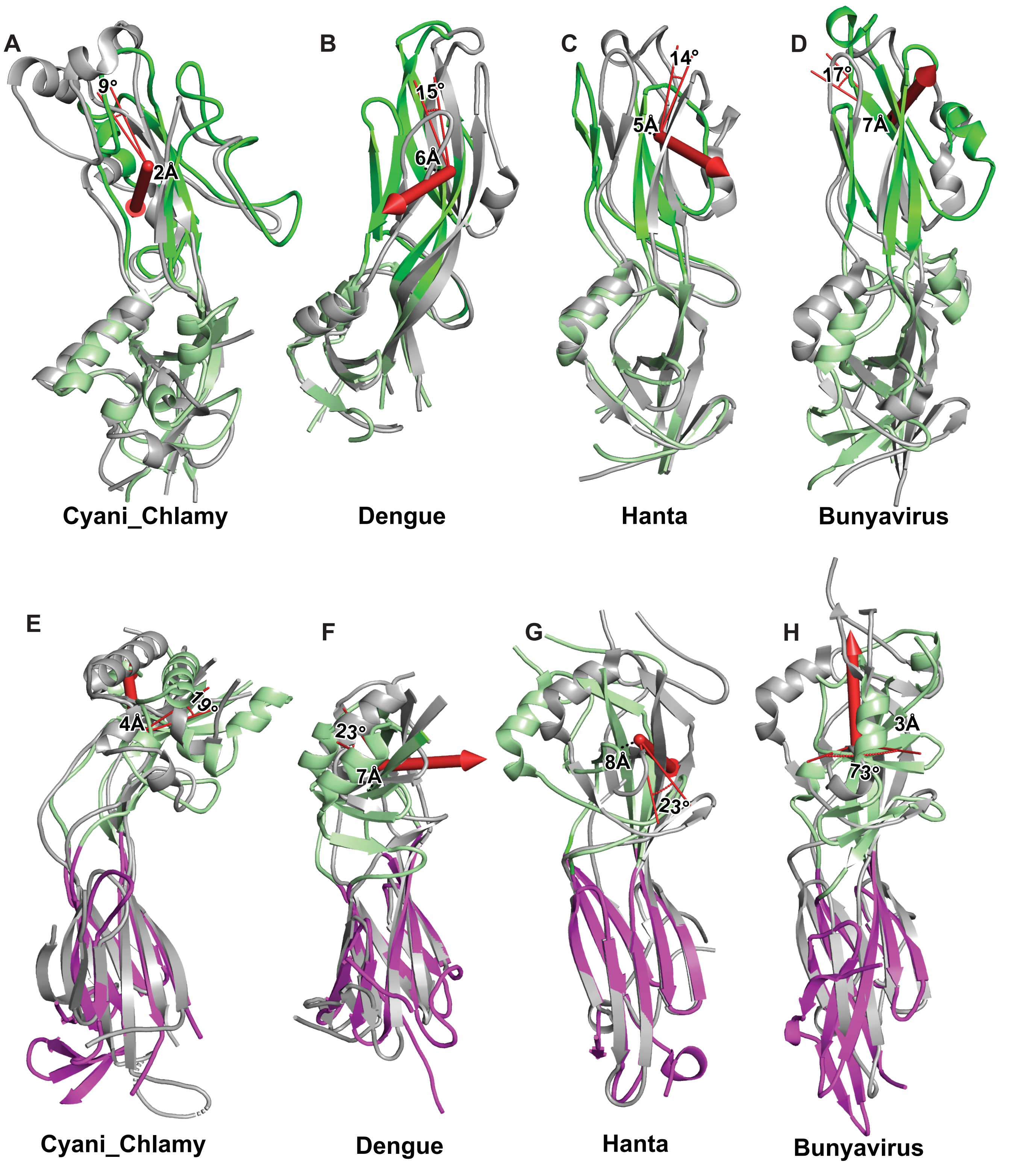

Supplementary Figure 1. D2.1 and D2.2 re-orientation between pre- and postfusion states of HAP2 and Class II fusogens. (A-D) D2.2-D2.1 and (E-H) D2.1-D1 reorientation in HAP2 (A,E), Dengue (B,F), Hantavirus (C,G) and Bunyavirus (D,H). In prefusion structures D2.2 is colored green, D2.1 is colored light green, and D1 is colored magenta. Post-fusion structures are colored silver. PDB files and methods are the same as described in the Figure 4 legend.

A 26 h at pH 2.0 in β-DDM. Box diameter 252Å, Mask diameter 250Å, 8696 particles

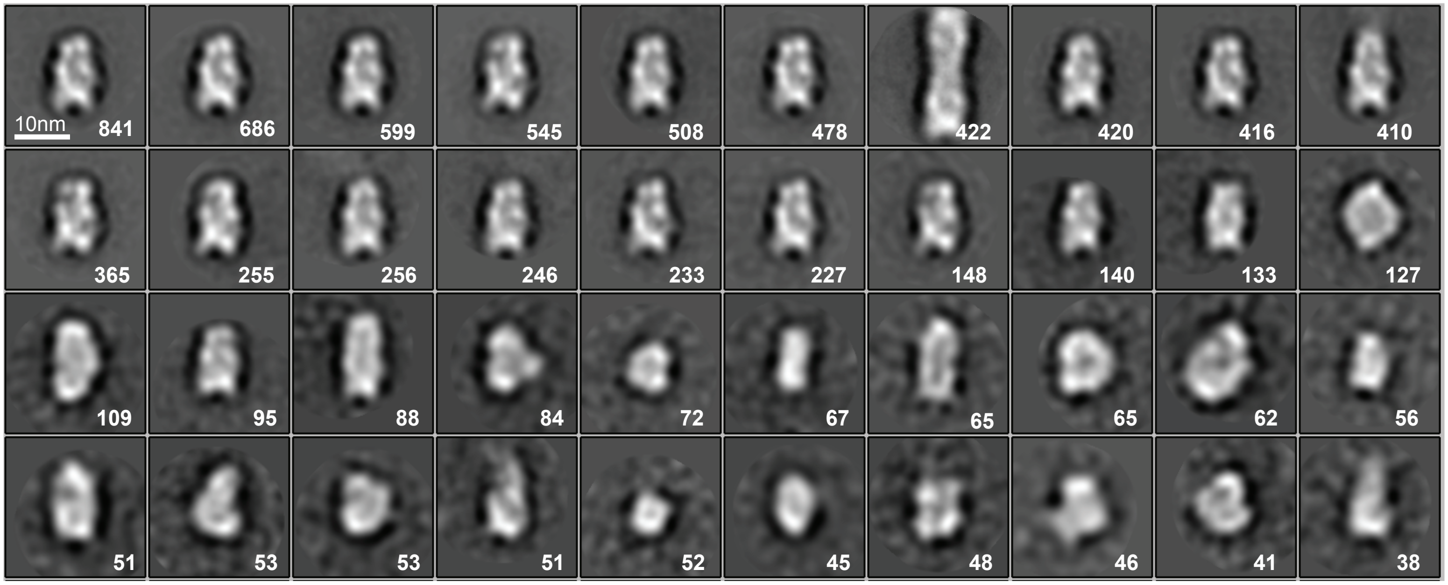

B pH 7.5, Box diameter 426Å, mask diameter 380Å, 1985 particles

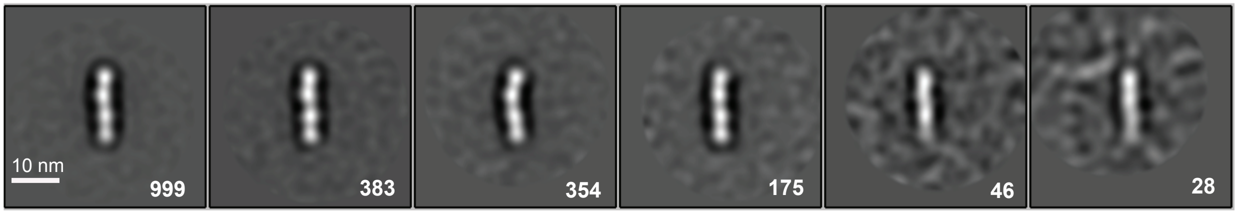

C pH 7.5, β-DDM 2h, Box diameter 426Å, mask diameter 400 Å, 2354 particles

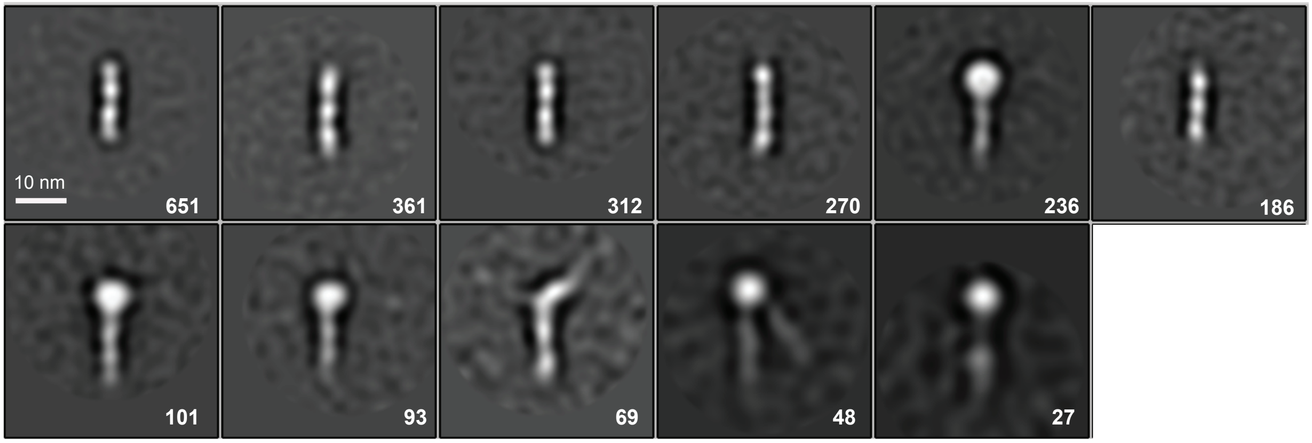

Supplementary Figure 2. Negative stain EM class averages of Cyani HAP2 ectodomain. (A-C) Class averages of Cyani HAP2 after incubation at 37°C as shown above each panel.

A pH 5.0, 0.1% β-DDM, 2 h; box diameter 426Å, mask diameter 400 Å, 2836 particles

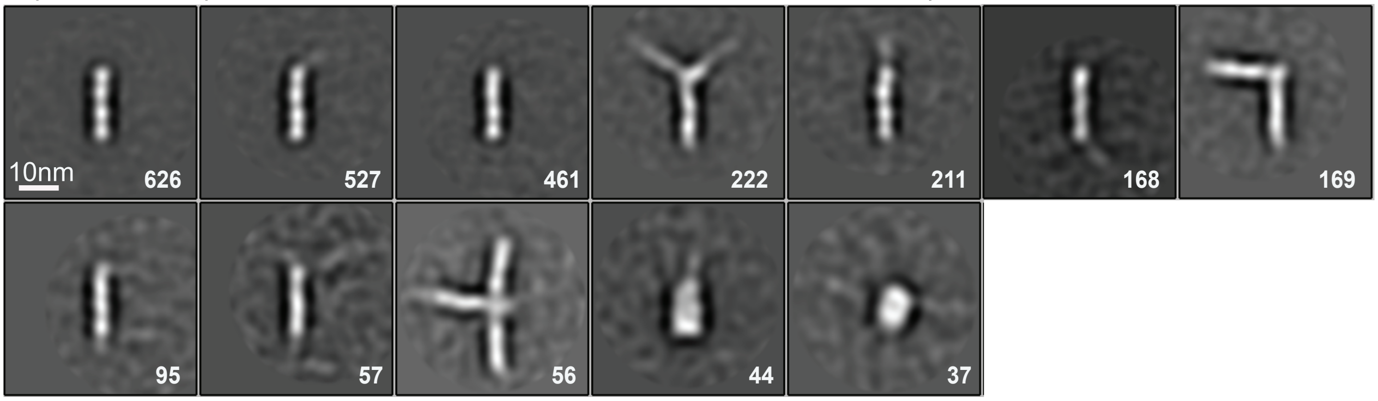

B pH 5.0, 0.1% β-DDM, 26 h, box diameter 320Å, mask diameter 300 Å, 4569 particles

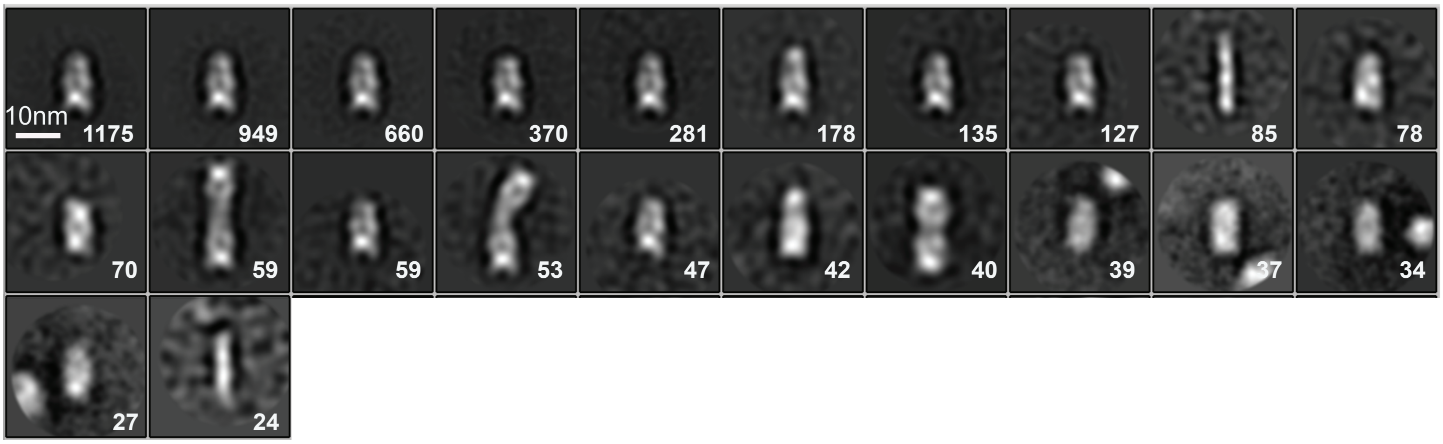

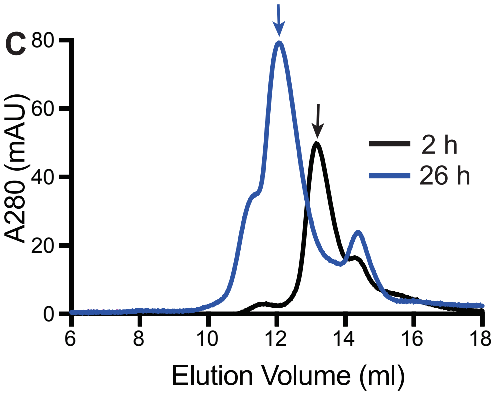

Supplementary Figure 3. Trimerization of Cyani HAP2 at pH 5.0 at different time points in β-DDM. (A-B) Negative stain EM class averages after incubation at 37°C as shown above each panel. (C) Size exclusion chromatography of Cyani HAP2 preparations mixed with 0.1% β-DDM for varying times at 37°C at pH 5.0 and subjected to Superdex 200 chromatography. Arrows mark peaks subjected to EM.

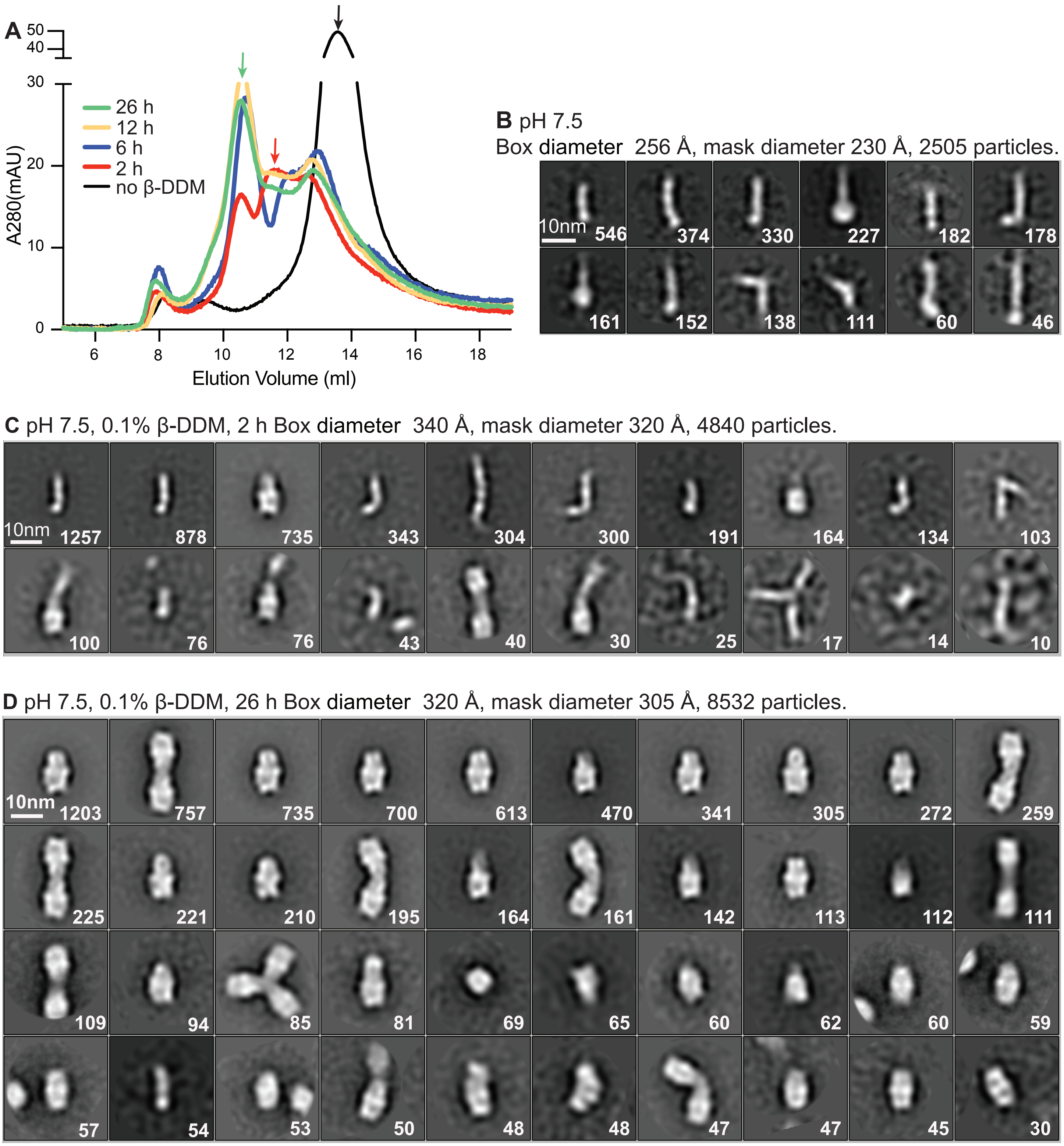

Supplementary Figure 4. EM class averages of Chlamy HAP2 at pH 7.5 with or without β-DDM at 23°C. (A) Superdex S200 size exclusion chromatography of Chlamy HAP2 preparations mixed with β-DDM for different times. Arrows show fractions used in EM. (B-D) Negative stain EM class averages of Chlamy HAP2 after incubation at 23°C as shown above each panel.

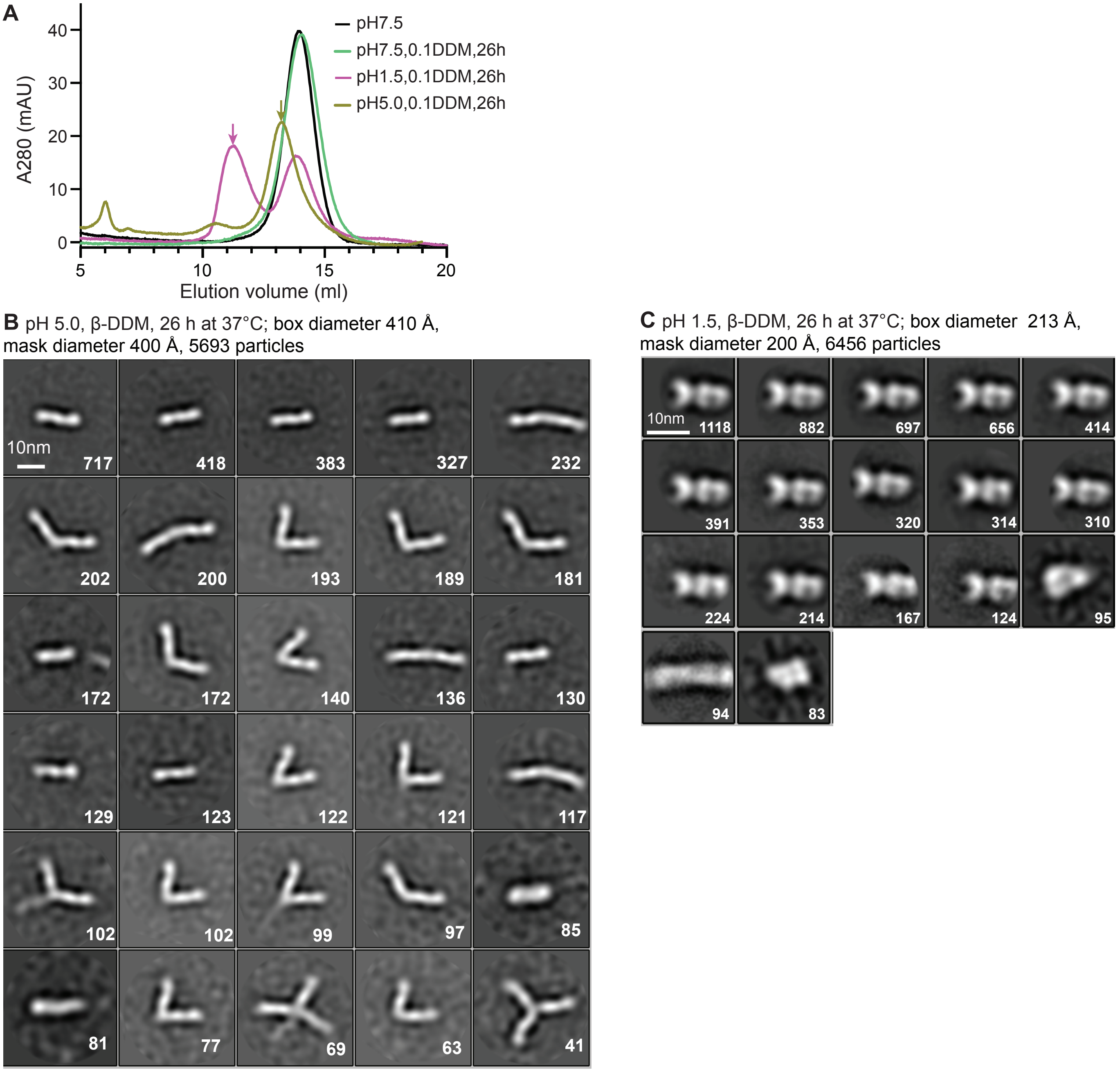

Supplementary Figure 5. Negative stain EM class averages of Cyani HAP2 D1D2. (A) Size exclusion chromatography of Cyani HAP2 D1D2 preparations treated with or without β-DDM at the indicated pH values and times at 37°C. Arrows show peak fractions taken for negative stain EM. (B-C) Class averages of Cyani HAP2 D1D2 after incubation at 37°C at the indicated pH and time. (B) pH 5.0 after 26 h. (C) pH 1.5 after 26 h.

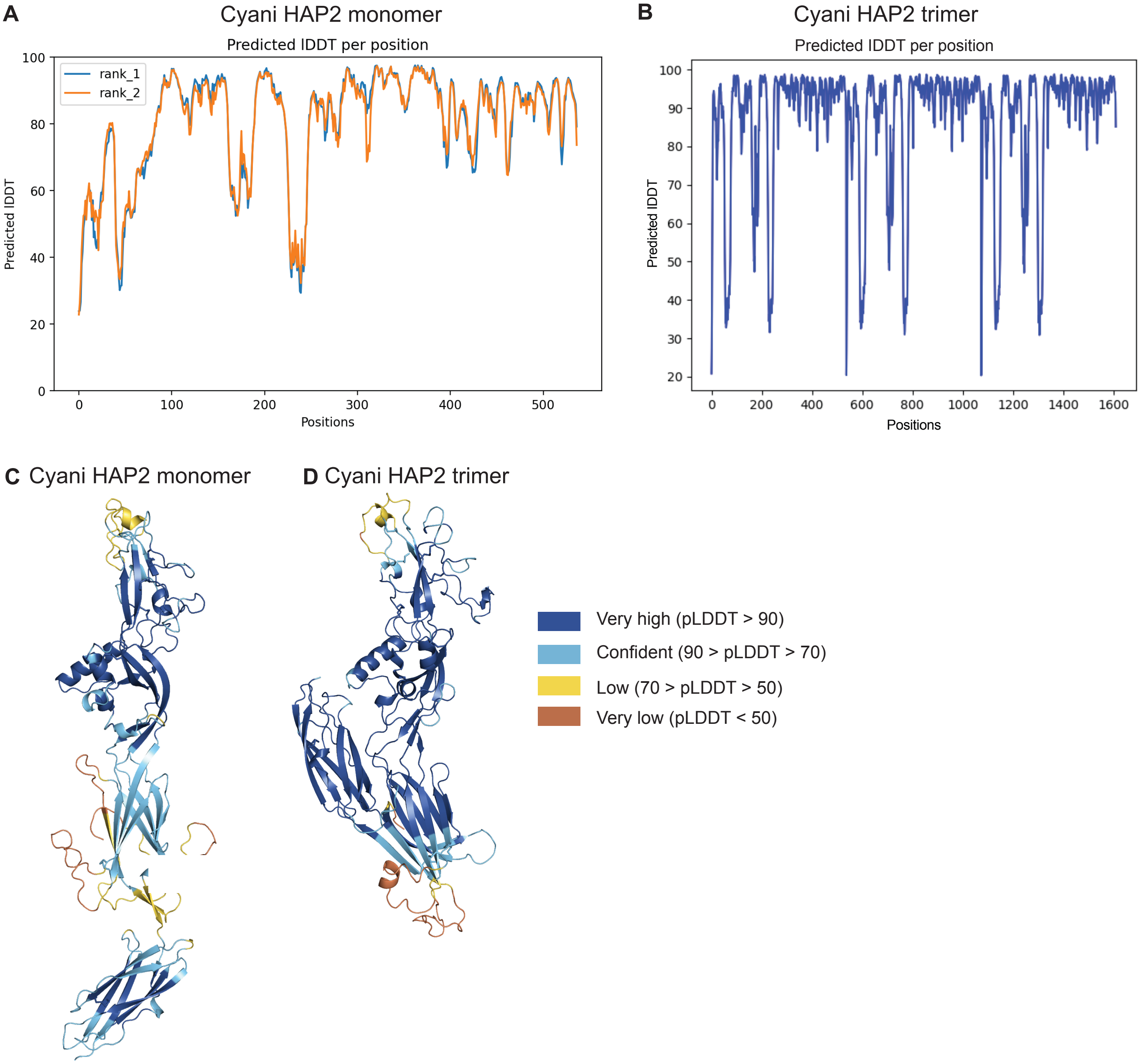

Supplementary Figure 6. Reliablity of AlphaFold predictions. (A-D) Predicted local-distance difference test (pLDDT) values plotted by sequence (A,B) and on the structures (C,D). (A) The pLDDT scores for two models of the Cyani HAP2 monomer. (B) The highest scoring Cyani HAP2 trimer AlphaFold-Multimer model. (C) The predicted Cyani HAP2 monomer. (D) one monomer from the Cyani HAP2 trimer chain predicted with AlphaFold-Multimer. The Cyani HAP2 monomeric was predicted using https://colab.research.google.com/github/sokrypton/ColabFold/blob/main/beta/AlphaFold2_advanced.ipynb. The Cyani HAP2 trimer was predicted with AlphaFold-Multimer (1.0) on the cosmic server (https://cosmic2.sdsc.edu:8443/gateway/home.action). Models with the highest pLDDT score were chosen.

Supplementary Table 1. Statistics of X-ray diffraction and structure refinement

	CmeHAP2
**Data collection statistics**	
Space group	$C222_1$
α, β, γ, °	90, 90, 90
Unit cell (a, b, c), Å	102.96, 197.01, 74.18
Resolution range (Å)	50.0-2.30 (2.36-2.30) [a]
Completeness (%)	98.8 (94.5)
Number unique reflections	33,534 (2,329)
Redundancy	3.3 (3.2)
R_{merge} (%) [b]	12.2 (242.6)
$I/\sigma(I)$	7.11(0.45)
$CC_{1/2}$ (%)[c]	99.3(22.5)
Wavelength (Å)	1.0332
**Refinement statistics**	
R_{work} (%)[d]	22.32 (39.81)
R_{free} (%)	26.25 (42.54)
Bond RMSD (Å)	0.005
Angle RMSD (°)	0.825
Ramachandran plot[e]	
(Favored/allowed/outlier)	95.61/4.18/0.21
Number of atoms	
Protein	3759
Ligand	105
Water	72
B factor	
Protein	87.93
Ligand	129.74
Water	75.31
Molprobity percentile	
(Clash/Geometry)	100/99
PDB	7S0K

[a] The numbers in parentheses refer to the highest resolution shell.

[b] Rmerge = $(\Sigma h\ \Sigma i\ |Ii(h) - \langle I(h)\rangle|) / \Sigma h \Sigma i\ Ii(h)$, where Ii(h) and <I(h)> are the ith and mean measurement of the intensity of reflection h.

[c] Pearson's correlation coefficient between average intensities of random half-datasets for each unique reflection (Karplus, P. A. and K. Diederichs (2012). "Linking crystallographic model and data quality." Science 336(6084): 1030-1033).

[d] Rfactor = $(\Sigma h||Fobs\ (h)|-|Fcalc\ (h)||) / \Sigma h|Fobs\ (h)|$, where Fobs (h) and F calc (h) are the observed and calculated structure factors, respectively. No $I/\sigma(I)$ cutoff was applied.

[e] Calculated with MolProbity: Davis, I. W., et al. (2007). "MolProbity: all-atom contacts and structure validation for proteins and nucleic acids." Nucleic Acids Res. 35: W375-383.
