# Supplementary material for: Monomeric prefusion structure of an extremophile gamete fusogen and stepwise formation of the postfusion trimeric state
Source: Nat Commun. 2022 Jul 13;13:4064. doi: 10.1038/s41467-022-31744-z (PMC9279424; doi:10.1038/s41467-022-31744-z)
Supplement: Supplementary file 3 — Reporting Summary [file 41467_2022_31744_MOESM3_ESM.pdf]

nature portfolio

Corresponding author(s): Timothy A Springer

Last updated by author(s): 6/23/2022

Reporting Summary

Nature Portfolio wishes to improve the reproducibility of the work that we publish. This form provides structure for consistency and transparency in reporting. For further information on Nature Portfolio policies, see our Editorial Policies and the Editorial Policy Checklist.

Statistics

For all statistical analyses, confirm that the following items are present in the figure legend, table legend, main text, or Methods section.

n/a	Confirmed	
☒	☐	The exact sample size (n) for each experimental group/condition, given as a discrete number and unit of measurement
☒	☐	A statement on whether measurements were taken from distinct samples or whether the same sample was measured repeatedly
☒	☐	The statistical test(s) used AND whether they are one- or two-sided<br>*Only common tests should be described solely by name; describe more complex techniques in the Methods section.*
☒	☐	A description of all covariates tested
☒	☐	A description of any assumptions or corrections, such as tests of normality and adjustment for multiple comparisons
☒	☐	A full description of the statistical parameters including central tendency (e.g. means) or other basic estimates (e.g. regression coefficient) AND variation (e.g. standard deviation) or associated estimates of uncertainty (e.g. confidence intervals)
☒	☐	For null hypothesis testing, the test statistic (e.g. F, t, r) with confidence intervals, effect sizes, degrees of freedom and P value noted<br>*Give P values as exact values whenever suitable.*
☒	☐	For Bayesian analysis, information on the choice of priors and Markov chain Monte Carlo settings
☒	☐	For hierarchical and complex designs, identification of the appropriate level for tests and full reporting of outcomes
☒	☐	Estimates of effect sizes (e.g. Cohen's d, Pearson's r), indicating how they were calculated

Our web collection on statistics for biologists contains articles on many of the points above.

Software and code

Policy information about availability of computer code

Data collection	Diffraction data of crystal were collected at 100K on GM/CA beamline 23-IDB at the Advanced Photon Source (Argonne National Laboratory). Images of negative-stain EM were acquired on an FEI Tecnai-12 transmission electron microscope at 120 KV.
Data analysis	Diffraction data of crystal were processed with XDS (Version June 1, 2017 BUILT=20170601). The structure was solved by molecular replacement with MR-Rosetta in the Phenix suite(1.20.1-4487-000) using PDB ID 6DBS as search model. Structures were refined with PHENIX, built with Coot (0.9.8.1 EL (ccp4)). Particles of negative stain EM were picked interactively and subjected to 2D alignment, classification and averaging using RELION 3.1. Alignment of class averages with one another, centering, and cross-correlation with crystal structures was done using EMAN2. Alphafold(https://colab.research.google.com/github/sokrypton/ColabFold/blob/main/beta/AlphaFold2_advanced.ipynb) and AlphaFold-Multimer (https://github.com/deepmind/AlphaFold) was used to predict the monomeric and trimeric state of Cyani HAP2.

For manuscripts utilizing custom algorithms or software that are central to the research but not yet described in published literature, software must be made available to editors and reviewers. We strongly encourage code deposition in a community repository (e.g. GitHub). See the Nature Portfolio guidelines for submitting code & software for further information.

Data

Policy information about availability of data

All manuscripts must include a data availability statement. This statement should provide the following information, where applicable:

- Accession codes, unique identifiers, or web links for publicly available datasets
- A description of any restrictions on data availability
- For clinical datasets or third party data, please ensure that the statement adheres to our policy

The atomic coordinates and the structure factors for Cyani HAP2 have been deposited in the PDB under the accession number 7S0K (https://www.rcsb.org/

structure/7S0K). Monomeric and trimeric Cyani HAP2 structures predicted with AlphaFold have been uploaded to Zenodo (https://zenodo.org/record/6476359#.YmG2IpPMJfq) under the Zenodo DOI Badge (10.5281/zenodo.6476359).

Field-specific reporting

Please select the one below that is the best fit for your research. If you are not sure, read the appropriate sections before making your selection.

- [x] Life sciences
- [ ] Behavioural & social sciences
- [ ] Ecological, evolutionary & environmental sciences

For a reference copy of the document with all sections, see nature.com/documents/nr-reporting-summary-flat.pdf

Life sciences study design

All studies must disclose on these points even when the disclosure is negative.

Sample size	No sample size calculations were performed because no statistical analysis was applied to our study. The preparations(protein) treated at different conditions and different time points are shown in each figure and figure legend.
Data exclusions	No data were excluded from the analysis.
Replication	All experiments were repeated at least twice with essentially identical results.
Randomization	This section does not apply to our study, given that no experimental groups were used, and no randomization of the data was performed.
Blinding	This section does not apply to our study.

Reporting for specific materials, systems and methods

We require information from authors about some types of materials, experimental systems and methods used in many studies. Here, indicate whether each material, system or method listed is relevant to your study. If you are not sure if a list item applies to your research, read the appropriate section before selecting a response.

Materials & experimental systems

n/a	Involved in the study	
x		Antibodies
	x	Eukaryotic cell lines
x		Palaeontology and archaeology
x		Animals and other organisms
x		Human research participants
x		Clinical data
x		Dual use research of concern

Methods

n/a	Involved in the study	
x		ChIP-seq
x		Flow cytometry
x		MRI-based neuroimaging

Eukaryotic cell lines

Policy information about cell lines

Cell line source(s)	Expi293F cells (Thermo Fisher Scientific)and Drosophila Schneider S2 cells(ExpreS2 cells, ExpreS2ion Biotechnologies) were used to express the protein(mentioned in material and methods).
Authentication	The cell lines were not authenticated.
Mycoplasma contamination	All cell lines test negative for Mycoplasma contamination.
Commonly misidentified lines (See ICLAC register)	No commonly misidentified cell lines were used in the study.
