# Supplementary material for: Monomeric prefusion structure of an extremophile gamete fusogen and stepwise formation of the postfusion trimeric state
Source: Nat Commun. 2022 Jul 13;13:4064. doi: 10.1038/s41467-022-31744-z (PMC9279424; doi:10.1038/s41467-022-31744-z)
Supplement: Supplementary file 4 — Source Data [file 41467_2022_31744_MOESM4_ESM.zip › NC_Source-Data_NCOMMS-21-43412B/7S0K_D_1000259368_val-report-full-annotate_P1.pdf]

Full wwPDB X-ray Structure Validation Report (i)

Aug 31, 2021 – 10:13 AM EDT

PDB ID	:	7S0K
Title	:	Crystal structure of the protein M
Deposited on	:	2021-08-30
Resolution	:	2.30 Å(reported)

This is a Full wwPDB X-ray Structure Validation Report.

This report is produced by the wwPDB biocuration pipeline after annotation of the structure.

We welcome your comments at *validation@mail.wwpdb.org*
A user guide is available at
https://www.wwpdb.org/validation/2017/XrayValidationReportHelp
with specific help available everywhere you see the (i) symbol.

CONFIDENTIAL VALIDATION REPORT

The following versions of software and data (see references (i)) were used in the production of this report:

MolProbity	:	4.02b-467
Mogul	:	1.8.5 (274361), CSD as541be (2020)
Xtriage (Phenix)	:	1.13
EDS	:	2.23.1
Percentile statistics	:	20191225.v01 (using entries in the PDB archive December 25th 2019)
Refmac	:	5.8.0158
CCP4	:	7.0.044 (Gargrove)
Ideal geometry (proteins)	:	Engh & Huber (2001)
Ideal geometry (DNA, RNA)	:	Parkinson et al. (1996)
Validation Pipeline (wwPDB-VP)	:	2.23.1

1 Overall quality at a glance (i)

The following experimental techniques were used to determine the structure:
X-RAY DIFFRACTION

The reported resolution of this entry is 2.30 Å.

Percentile scores (ranging between 0-100) for global validation metrics of the entry are shown in the following graphic. The table shows the number of entries on which the scores are based.

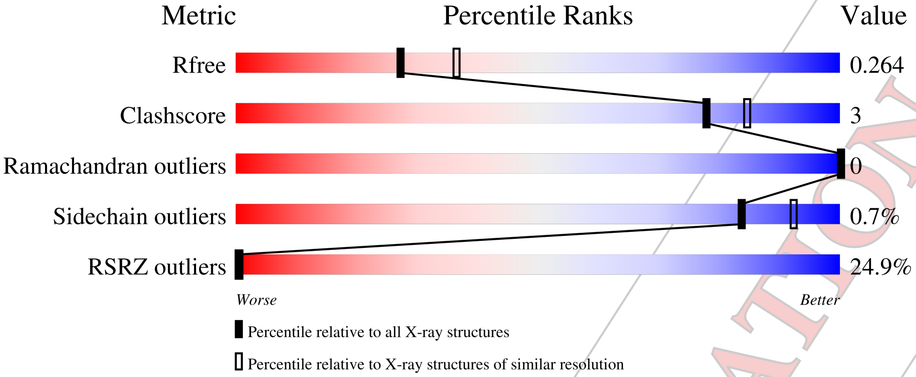

Metric	Whole archive (#Entries)	Similar resolution (#Entries, resolution range(Å))
R_{free}	130704	5042 (2.30-2.30)
Clashscore	141614	5643 (2.30-2.30)
Ramachandran outliers	138981	5575 (2.30-2.30)
Sidechain outliers	138945	5575 (2.30-2.30)
RSRZ outliers	127900	4938 (2.30-2.30)

The table below summarises the geometric issues observed across the polymeric chains and their fit to the electron density. The red, orange, yellow and green segments of the lower bar indicate the fraction of residues that contain outliers for >=3, 2, 1 and 0 types of geometric quality criteria respectively. A grey segment represents the fraction of residues that are not modelled. The numeric value for each fraction is indicated below the corresponding segment, with a dot representing fractions <=5% The upper red bar (where present) indicates the fraction of residues that have poor fit to the electron density. The numeric value is given above the bar.

Mol	Chain	Length	Quality of chain
1	A	545	22% / 82% 7% 11%
2	B	5	20% 80%

The following table lists non-polymeric compounds, carbohydrate monomers and non-standard residues in protein, DNA, RNA chains that are outliers for geometric or electron-density-fit criteria:

CONFIDENTIAL VALIDATION REPORT

Mol	Type	Chain	Res	Chirality	Geometry	Clashes	Electron density
2	BMA	B	4	-	-	-	X
2	MAN	B	5	-	-	-	X
3	NAG	A	601	-	-	-	X

CONFIDENTIAL VALIDATION REPORT

2 Entry composition (i)

There are 6 unique types of molecules in this entry. The entry contains 3930 atoms, of which 0 are hydrogens and 0 are deuteriums.

In the tables below, the ZeroOcc column contains the number of atoms modelled with zero occupancy, the AltConf column contains the number of residues with at least one atom in alternate conformation and the Trace column contains the number of residues modelled with at most 2 atoms.

- Molecule 1 is a protein called HAP2-GCS1 domain-containing protein.

Mol	Chain	Residues	Atoms					ZeroOcc	AltConf	Trace
			Total	C	N	O	S			
1	A	485	3750	2379	619	732	20	0	0	0

There are 8 discrepancies between the modelled and reference sequences:

Chain	Residue	Modelled	Actual	Comment	Reference
A	565	HIS	-	expression tag	UNP M1V8A6
A	566	HIS	-	expression tag	UNP M1V8A6
A	567	HIS	-	expression tag	UNP M1V8A6
A	568	HIS	-	expression tag	UNP M1V8A6
A	569	HIS	-	expression tag	UNP M1V8A6
A	570	HIS	-	expression tag	UNP M1V8A6
A	571	HIS	-	expression tag	UNP M1V8A6
A	572	HIS	-	expression tag	UNP M1V8A6

- Molecule 2 is an oligosaccharide called alpha-D-mannopyranose-(1-3)-beta-D-mannopyranose-(1-3)-beta-D-mannopyranose-(1-4)-2-acetamido-2-deoxy-beta-D-glucopyranose-(1-4)-2-acetamido-2-deoxy-beta-D-glucopyranose.

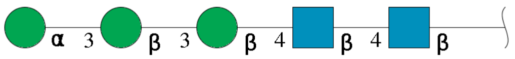

Mol	Chain	Residues	Atoms				ZeroOcc	AltConf	Trace
			Total	C	N	O			
2	B	5	61	34	2	25	0	0	0

- Molecule 3 is 2-acetamido-2-deoxy-beta-D-glucopyranose (three-letter code: NAG) (formula: $C_8H_{15}NO_6$).

CONFIDENTIAL VALIDATION REPORT

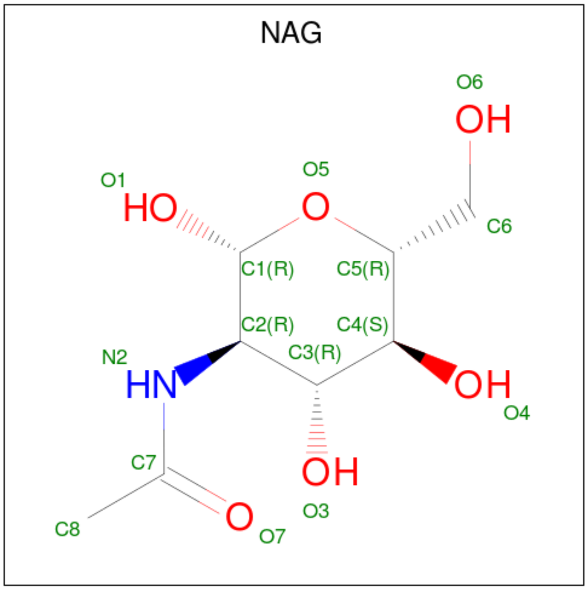

Mol	Chain	Residues	Atoms	ZeroOcc	AltConf
3	A	1	Total C N O<br>14 8 1 5	0	0

- Molecule 4 is GLYCEROL (three-letter code: GOL) (formula: $C_3H_8O_3$).

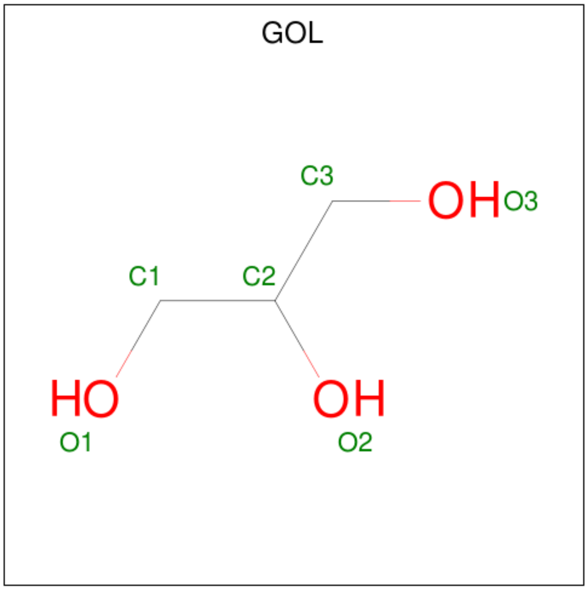

Mol	Chain	Residues	Atoms	ZeroOcc	AltConf
4	A	1	Total C O<br>6 3 3	0	0
4	A	1	Total C O<br>6 3 3	0	0
4	A	1	Total C O<br>6 3 3	0	0

CONFIDENTIAL VALIDATION REPORT

- Molecule 5 is DI(HYDROXYETHYL)ETHER (three-letter code: PEG) (formula: $C_4H_{10}O_3$).

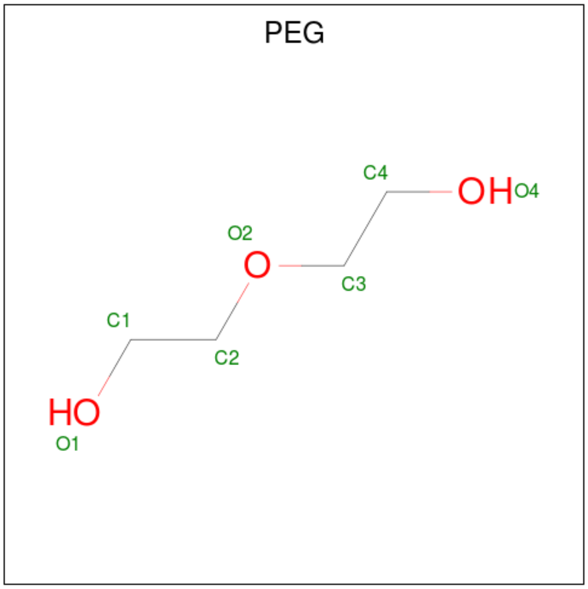

Mol	Chain	Residues	Atoms	ZeroOcc	AltConf
5	A	1	Total C O<br>7 4 3	0	0
5	A	1	Total C O<br>7 4 3	0	0

- Molecule 6 is water.

Mol	Chain	Residues	Atoms	ZeroOcc	AltConf
6	A	73	Total O<br>73 73	0	0

CONFIDENTIAL VALIDATION REPORT

3 Residue-property plots ⓘ

These plots are drawn for all protein, RNA, DNA and oligosaccharide chains in the entry. The first graphic for a chain summarises the proportions of the various outlier classes displayed in the second graphic. The second graphic shows the sequence view annotated by issues in geometry and electron density. Residues are color-coded according to the number of geometric quality criteria for which they contain at least one outlier: green = 0, yellow = 1, orange = 2 and red = 3 or more. A red dot above a residue indicates a poor fit to the electron density (RSRZ > 2). Stretches of 2 or more consecutive residues without any outlier are shown as a green connector. Residues present in the sample, but not in the model, are shown in grey.

- Molecule 1: HAP2-GCS1 domain-containing protein

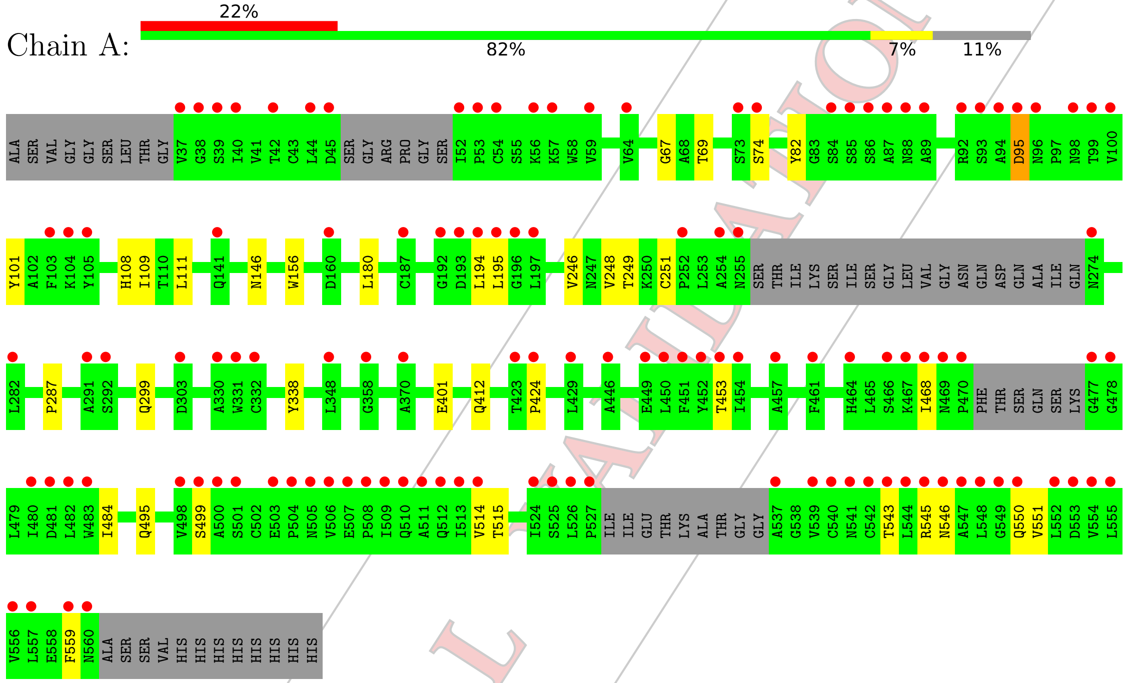

- Molecule 2: alpha-D-mannopyranose-(1-3)-beta-D-mannopyranose-(1-3)-beta-D-mannopyranos e-(1-4)-2-acetamido-2-deoxy-beta-D-glucopyranose-(1-4)-2-acetamido-2-deoxy-beta-D-glucopyran ose

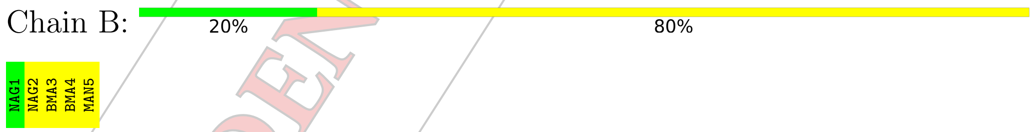

CONFIDENTIAL VALIDATION REPORT

4 Data and refinement statistics (i)

Property	Value	Source
Space group	C 2 2 21	Depositor
Cell constants<br>a, b, c, α, β, γ	102.96Å 197.01Å 74.18Å<br>90.00° 90.00° 90.00°	Depositor
Resolution (Å)	45.63 – 2.30<br>45.63 – 2.30	Depositor<br>EDS
% Data completeness<br>(in resolution range)	98.8 (45.63-2.30)<br>98.8 (45.63-2.30)	Depositor<br>EDS
R_{merge}	(Not available)	Depositor
R_{sym}	(Not available)	Depositor
$< I/\sigma(I) >$ [1]	0.97 (at 2.29Å)	Xtriage
Refinement program	PHENIX 1.17.1_3660	Depositor
R, R_{free}	0.223 , 0.264<br>0.223 , 0.264	Depositor<br>DCC
R_{free} test set	1695 reflections (5.06%)	wwPDB-VP
Wilson B-factor (Å^2)	66.7	Xtriage
Anisotropy	0.260	Xtriage
Bulk solvent k_{sol}(e/Å^3), B_{sol}(Å^2)	0.31 , 56.8	EDS
L-test for twinning[2]	$< \|L\| > = 0.49$, $< L^2 > = 0.32$	Xtriage
Estimated twinning fraction	No twinning to report.	Xtriage
F_o,F_c correlation	0.95	EDS
Total number of atoms	3930	wwPDB-VP
Average B, all atoms (Å^2)	90.0	wwPDB-VP

Xtriage's analysis on translational NCS is as follows: *The largest off-origin peak in the Patterson function is 4.02% of the height of the origin peak. No significant pseudotranslation is detected.*

[1] Intensities estimated from amplitudes.

[2] Theoretical values of $< |L| >$, $< L^2 >$ for acentric reflections are 0.5, 0.333 respectively for untwinned datasets, and 0.375, 0.2 for perfectly twinned datasets.

CONFIDENTIAL VALIDATION REPORT

5 Model quality (i)

5.1 Standard geometry (i)

Bond lengths and bond angles in the following residue types are not validated in this section: GOL, PEG, NAG, MAN, BMA

The Z score for a bond length (or angle) is the number of standard deviations the observed value is removed from the expected value. A bond length (or angle) with $|Z| > 5$ is considered an outlier worth inspection. RMSZ is the root-mean-square of all Z scores of the bond lengths (or angles).

Mol	Chain	Bond lengths		Bond angles	
		RMSZ	#$\|Z\|$ >5	RMSZ	#$\|Z\|$ >5
1	A	0.26	0/3838	0.46	0/5240

There are no bond length outliers.

There are no bond angle outliers.

There are no chirality outliers.

There are no planarity outliers.

5.2 Too-close contacts (i)

In the following table, the Non-H and H(model) columns list the number of non-hydrogen atoms and hydrogen atoms in the chain respectively. The H(added) column lists the number of hydrogen atoms added and optimized by MolProbity. The Clashes column lists the number of clashes within the asymmetric unit, whereas Symm-Clashes lists symmetry-related clashes.

Mol	Chain	Non-H	H(model)	H(added)	Clashes	Symm-Clashes
1	A	3750	0	3596	22	0
2	B	61	0	52	1	0
3	A	14	0	13	0	0
4	A	18	0	24	2	0
5	A	14	0	20	0	0
6	A	73	0	0	1	0
All	All	3930	0	3705	22	0

The all-atom clashscore is defined as the number of clashes found per 1000 atoms (including hydrogen atoms). The all-atom clashscore for this structure is 3.

All (22) close contacts within the same asymmetric unit are listed below, sorted by their clash magnitude.

CONFIDENTIAL VALIDATION REPORT

Atom-1	Atom-2	Interatomic distance (Å)	Clash overlap (Å)
1:A:546:ASN:ND2	1:A:550:GLN:OE1	2.23	0.70
1:A:95:ASP:OD1	1:A:95:ASP:N	2.27	0.65
1:A:67:GLY:H	4:A:604:GOL:H32	1.62	0.64
1:A:546:ASN:HB2	1:A:550:GLN:HB2	1.82	0.61
1:A:401:GLU:OE1	1:A:412:GLN:NE2	2.34	0.61
1:A:109:ILE:HG22	1:A:248:VAL:HG22	1.85	0.58
1:A:338:TYR:CZ	1:A:424:PRO:HA	2.42	0.55
1:A:82:TYR:HE2	1:A:109:ILE:HD11	1.71	0.55
1:A:495:GLN:HG2	1:A:515:THR:HG22	1.88	0.55
1:A:499:SER:OG	1:A:543:THR:OG1	2.25	0.54
1:A:111:LEU:HD22	1:A:246:VAL:HG22	1.91	0.53
1:A:545:ARG:HG2	1:A:551:VAL:HG22	1.89	0.53
1:A:108:HIS:HB2	1:A:249:THR:HB	1.93	0.50
1:A:69:THR:HB	1:A:74:SER:HA	1.94	0.48
1:A:468:ILE:HG21	1:A:559:PHE:HD1	1.80	0.47
1:A:101:TYR:CE2	1:A:453:THR:HG22	2.50	0.46
1:A:251:CYS:SG	2:B:2:NAG:H81	2.56	0.46
1:A:484:ILE:HD11	1:A:514:VAL:HG11	1.98	0.45
1:A:287:PRO:O	1:A:299:GLN:HA	2.19	0.43
1:A:67:GLY:O	4:A:604:GOL:H32	2.19	0.43
1:A:156:TRP:CZ3	1:A:180:LEU:HA	2.56	0.41
1:A:146:ASN:ND2	6:A:702:HOH:O	2.54	0.41

There are no symmetry-related clashes.

5.3 Torsion angles (i)

5.3.1 Protein backbone (i)

In the following table, the Percentiles column shows the percent Ramachandran outliers of the chain as a percentile score with respect to all X-ray entries followed by that with respect to entries of similar resolution.

The Analysed column shows the number of residues for which the backbone conformation was analysed, and the total number of residues.

Mol	Chain	Analysed	Favoured	Allowed	Outliers	Percentiles
1	A	475/545 (87%)	454 (96%)	21 (4%)	0	100 100

There are no Ramachandran outliers to report.

CONFIDENTIAL VALIDATION REPORT

5.3.2 Protein sidechains (i)

In the following table, the Percentiles column shows the percent sidechain outliers of the chain as a percentile score with respect to all X-ray entries followed by that with respect to entries of similar resolution.

The Analysed column shows the number of residues for which the sidechain conformation was analysed, and the total number of residues.

Mol	Chain	Analysed	Rotameric	Outliers	Percentiles
1	A	419/466 (90%)	416 (99%)	3 (1%)	84 92

All (3) residues with a non-rotameric sidechain are listed below:

Mol	Chain	Res	Type
1	A	95	ASP
1	A	194	LEU
1	A	195	LEU

Sometimes sidechains can be flipped to improve hydrogen bonding and reduce clashes. There are no such sidechains identified.

5.3.3 RNA (i)

There are no RNA molecules in this entry.

5.4 Non-standard residues in protein, DNA, RNA chains (i)

There are no non-standard protein/DNA/RNA residues in this entry.

5.5 Carbohydrates (i)

5 monosaccharides are modelled in this entry.

In the following table, the Counts columns list the number of bonds (or angles) for which Mogul statistics could be retrieved, the number of bonds (or angles) that are observed in the model and the number of bonds (or angles) that are defined in the Chemical Component Dictionary. The Link column lists molecule types, if any, to which the group is linked. The Z score for a bond length (or angle) is the number of standard deviations the observed value is removed from the expected value. A bond length (or angle) with $|Z| > 2$ is considered an outlier worth inspection. RMSZ is the root-mean-square of all Z scores of the bond lengths (or angles).

CONFIDENTIAL VALIDATION REPORT

Mol	Type	Chain	Res	Link	Bond lengths			Bond angles		
					Counts	RMSZ	#|Z| > 2	Counts	RMSZ	#|Z| > 2
2	NAG	B	1	2,1	14,14,15	0.38	0	17,19,21	0.55	0
2	NAG	B	2	2	14,14,15	0.36	0	17,19,21	0.56	0
2	BMA	B	3	2	11,11,12	1.29	2 (18%)	15,15,17	1.61	2 (13%)
2	BMA	B	4	2	11,11,12	1.27	3 (27%)	15,15,17	1.43	3 (20%)
2	MAN	B	5	2	11,11,12	1.23	2 (18%)	15,15,17	1.17	3 (20%)

In the following table, the Chirals column lists the number of chiral outliers, the number of chiral centers analysed, the number of these observed in the model and the number defined in the Chemical Component Dictionary. Similar counts are reported in the Torsion and Rings columns. '-' means no outliers of that kind were identified.

Mol	Type	Chain	Res	Link	Chirals	Torsions	Rings
2	NAG	B	1	2,1	-	2/6/23/26	0/1/1/1
2	NAG	B	2	2	-	2/6/23/26	0/1/1/1
2	BMA	B	3	2	-	0/2/19/22	0/1/1/1
2	BMA	B	4	2	-	2/2/19/22	0/1/1/1
2	MAN	B	5	2	-	0/2/19/22	0/1/1/1

All (7) bond length outliers are listed below:

Mol	Chain	Res	Type	Atoms	Z	Observed(Å)	Ideal(Å)
2	B	5	MAN	O5-C1	2.70	1.48	1.43
2	B	3	BMA	O5-C5	2.43	1.48	1.43
2	B	4	BMA	C2-C3	-2.35	1.49	1.52
2	B	5	MAN	C1-C2	2.34	1.57	1.52
2	B	3	BMA	C1-C2	-2.26	1.47	1.52
2	B	4	BMA	C4-C3	2.24	1.58	1.52
2	B	4	BMA	C4-C5	2.06	1.57	1.53

All (8) bond angle outliers are listed below:

Mol	Chain	Res	Type	Atoms	Z	Observed(°)	Ideal(°)
2	B	3	BMA	C1-C2-C3	-5.06	103.44	109.67
2	B	4	BMA	C1-O5-C5	2.83	116.03	112.19
2	B	4	BMA	O6-C6-C5	-2.56	102.50	111.29
2	B	4	BMA	O2-C2-C3	-2.54	105.05	110.14
2	B	5	MAN	O2-C2-C3	-2.23	105.68	110.14
2	B	5	MAN	C1-O5-C5	2.19	115.16	112.19
2	B	5	MAN	O5-C1-C2	2.18	114.14	110.77
2	B	3	BMA	O3-C3-C4	2.15	115.32	110.35

CONFIDENTIAL VALIDATION REPORT

There are no chirality outliers.

All (6) torsion outliers are listed below:

Mol	Chain	Res	Type	Atoms
2	B	1	NAG	O5-C5-C6-O6
2	B	2	NAG	O5-C5-C6-O6
2	B	4	BMA	C4-C5-C6-O6
2	B	2	NAG	C4-C5-C6-O6
2	B	1	NAG	C4-C5-C6-O6
2	B	4	BMA	O5-C5-C6-O6

There are no ring outliers.

1 monomer is involved in 1 short contact:

Mol	Chain	Res	Type	Clashes	Symm-Clashes
2	B	2	NAG	1	0

The following is a two-dimensional graphical depiction of Mogul quality analysis of bond lengths, bond angles, torsion angles, and ring geometry for oligosaccharide.

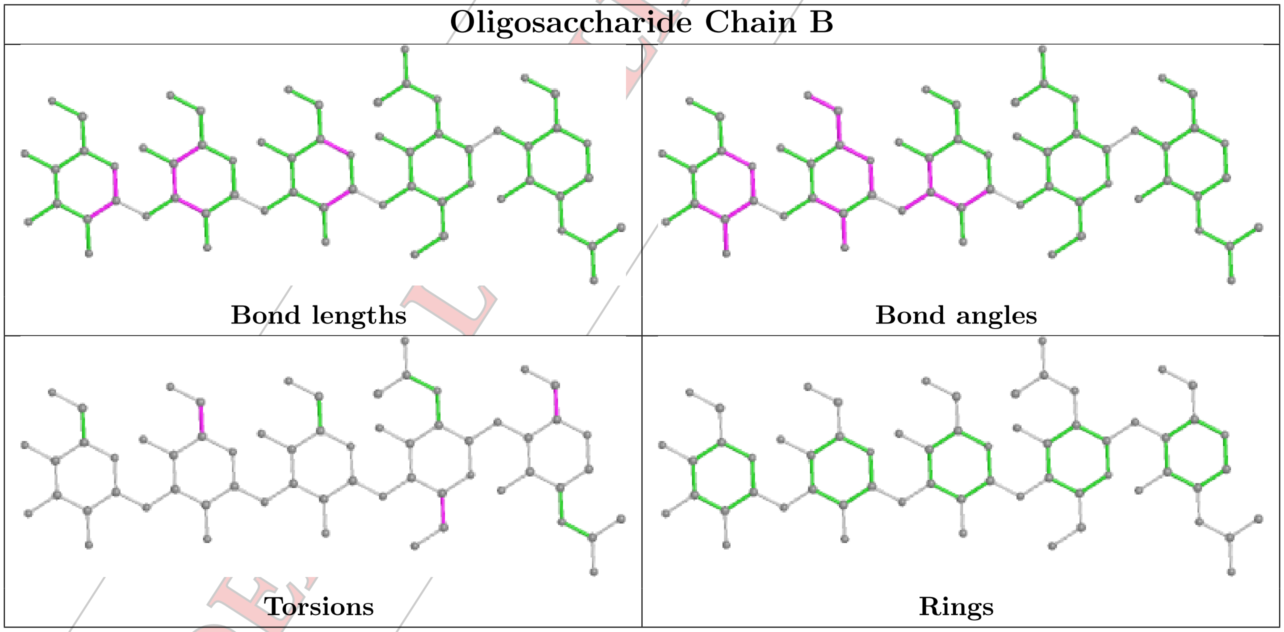

5.6 Ligand geometry (i)

6 ligands are modelled in this entry.

In the following table, the Counts columns list the number of bonds (or angles) for which Mogul statistics could be retrieved, the number of bonds (or angles) that are observed in the model and the number of bonds (or angles) that are defined in the Chemical Component Dictionary. The Link column lists molecule types, if any, to which the group is linked. The Z score for a bond

CONFIDENTIAL VALIDATION REPORT

length (or angle) is the number of standard deviations the observed value is removed from the expected value. A bond length (or angle) with $|Z| > 2$ is considered an outlier worth inspection. RMSZ is the root-mean-square of all Z scores of the bond lengths (or angles).

Mol	Type	Chain	Res	Link	Bond lengths			Bond angles		
					Counts	RMSZ	$\#\|Z\| > 2$	Counts	RMSZ	$\#\|Z\| > 2$
3	NAG	A	601	1	14,14,15	0.22	0	17,19,21	0.53	0
5	PEG	A	606	-	6,6,6	0.48	0	5,5,5	0.29	0
4	GOL	A	603	-	5,5,5	0.84	0	5,5,5	1.03	0
4	GOL	A	602	-	5,5,5	0.92	0	5,5,5	0.94	0
4	GOL	A	604	-	5,5,5	0.90	0	5,5,5	1.00	0
5	PEG	A	605	-	6,6,6	0.49	0	5,5,5	0.22	0

In the following table, the Chirals column lists the number of chiral outliers, the number of chiral centers analysed, the number of these observed in the model and the number defined in the Chemical Component Dictionary. Similar counts are reported in the Torsion and Rings columns. '-' means no outliers of that kind were identified.

Mol	Type	Chain	Res	Link	Chirals	Torsions	Rings
3	NAG	A	601	1	-	2/6/23/26	0/1/1/1
5	PEG	A	606	-	-	3/4/4/4	-
4	GOL	A	603	-	-	2/4/4/4	-
4	GOL	A	602	-	-	3/4/4/4	-
4	GOL	A	604	-	-	2/4/4/4	-
5	PEG	A	605	-	-	2/4/4/4	-

There are no bond length outliers.

There are no bond angle outliers.

There are no chirality outliers.

All (14) torsion outliers are listed below:

Mol	Chain	Res	Type	Atoms
4	A	602	GOL	O1-C1-C2-O2
4	A	602	GOL	O1-C1-C2-C3
4	A	603	GOL	O1-C1-C2-C3
3	A	601	NAG	O5-C5-C6-O6
4	A	604	GOL	O1-C1-C2-C3
5	A	606	PEG	O2-C3-C4-O4
3	A	601	NAG	C4-C5-C6-O6
4	A	603	GOL	O1-C1-C2-O2
5	A	605	PEG	C1-C2-O2-C3
5	A	605	PEG	C4-C3-O2-C2

Continued on next page...

CONFIDENTIAL VALIDATION REPORT

Continued from previous page...

Mol	Chain	Res	Type	Atoms
5	A	606	PEG	C1-C2-O2-C3
5	A	606	PEG	O1-C1-C2-O2
4	A	602	GOL	C1-C2-C3-O3
4	A	604	GOL	O1-C1-C2-O2

There are no ring outliers.

1 monomer is involved in 2 short contacts:

Mol	Chain	Res	Type	Clashes	Symm-Clashes
4	A	604	GOL	2	0

5.7 Other polymers (i)

There are no such residues in this entry.

5.8 Polymer linkage issues (i)

There are no chain breaks in this entry.

CONFIDENTIAL VALIDATION REPORT

6 Fit of model and data (i)

6.1 Protein, DNA and RNA chains (i)

In the following table, the column labelled '#RSRZ> 2' contains the number (and percentage) of RSRZ outliers, followed by percent RSRZ outliers for the chain as percentile scores relative to all X-ray entries and entries of similar resolution. The OWAB column contains the minimum, median, 95th percentile and maximum values of the occupancy-weighted average B-factor per residue. The column labelled 'Q< 0.9' lists the number of (and percentage) of residues with an average occupancy less than 0.9.

Mol	Chain	Analysed	<RSRZ>	#RSRZ>2			OWAB(Å^2)	Q<0.9
1	A	485/545 (88%)	1.80	121 (24%)	0	0	51, 79, 152, 193	0

All (121) RSRZ outliers are listed below:

Mol	Chain	Res	Type	RSRZ
1	A	478	GLY	12.4
1	A	549	GLY	12.2
1	A	559	PHE	11.9
1	A	508	PRO	11.7
1	A	524	ILE	11.1
1	A	192	GLY	10.8
1	A	193	ASP	10.2
1	A	194	LEU	10.1
1	A	509	ILE	8.8
1	A	88	ASN	8.6
1	A	477	GLY	7.8
1	A	498	VAL	7.7
1	A	87	ALA	7.6
1	A	548	LEU	7.5
1	A	527	PRO	7.5
1	A	510	GLN	7.0
1	A	550	GLN	6.9
1	A	99	THR	6.8
1	A	52	ILE	6.7
1	A	468	ILE	6.4
1	A	503	GLU	6.3
1	A	85	SER	6.1
1	A	556	VAL	6.0
1	A	195	LEU	5.8
1	A	542	CYS	5.7
1	A	94	ALA	5.7
1	A	546	ASN	5.6

Continued on next page...

CONFIDENTIAL VALIDATION REPORT

Continued from previous page...

Mol	Chain	Res	Type	RSRZ
1	A	482	LEU	5.4
1	A	525	SER	5.3
1	A	42	THR	5.1
1	A	541	ASN	5.1
1	A	555	LEU	5.0
1	A	499	SER	5.0
1	A	545	ARG	5.0
1	A	504	PRO	4.9
1	A	560	ASN	4.8
1	A	86	SER	4.8
1	A	95	ASP	4.8
1	A	507	GLU	4.7
1	A	96	ASN	4.7
1	A	547	ALA	4.5
1	A	74	SER	4.5
1	A	44	LEU	4.5
1	A	292	SER	4.5
1	A	543	THR	4.4
1	A	469	ASN	4.4
1	A	554	VAL	4.4
1	A	40	ILE	4.3
1	A	467	LYS	4.2
1	A	92	ARG	4.1
1	A	526	LEU	4.1
1	A	454	ILE	4.0
1	A	254	ALA	4.0
1	A	501	SER	4.0
1	A	100	VAL	3.9
1	A	511	ALA	3.9
1	A	552	LEU	3.7
1	A	557	LEU	3.7
1	A	98	ASN	3.7
1	A	38	GLY	3.7
1	A	466	SER	3.7
1	A	544	LEU	3.6
1	A	303	ASP	3.6
1	A	452	TYR	3.6
1	A	464	HIS	3.6
1	A	451	PHE	3.6
1	A	73	SER	3.5
1	A	513	ILE	3.3
1	A	37	VAL	3.3

Continued on next page...

CONFIDENTIAL VALIDATION REPORT

Continued from previous page...

Mol	Chain	Res	Type	RSRZ
1	A	450	LEU	3.3
1	A	481	ASP	3.3
1	A	45	ASP	3.2
1	A	54	CYS	3.2
1	A	105	TYR	3.1
1	A	480	ILE	3.1
1	A	500	ALA	3.1
1	A	483	TRP	3.1
1	A	540	CYS	3.1
1	A	470	PRO	3.1
1	A	537	ALA	3.0
1	A	274	ASN	3.0
1	A	539	VAL	2.9
1	A	93	SER	2.9
1	A	512	GLN	2.8
1	A	56	LYS	2.8
1	A	197	LEU	2.7
1	A	252	PRO	2.7
1	A	506	VAL	2.6
1	A	429	LEU	2.6
1	A	84	SER	2.6
1	A	39	SER	2.6
1	A	457	ALA	2.6
1	A	53	PRO	2.5
1	A	553	ASP	2.5
1	A	89	ALA	2.4
1	A	505	ASN	2.4
1	A	370	ALA	2.4
1	A	348	LEU	2.4
1	A	291	ALA	2.3
1	A	332	CYS	2.3
1	A	282	LEU	2.3
1	A	64	VAL	2.3
1	A	358	GLY	2.3
1	A	187	CYS	2.3
1	A	57	LYS	2.3
1	A	196	GLY	2.3
1	A	330	ALA	2.3
1	A	514	VAL	2.2
1	A	461	PHE	2.2
1	A	160	ASP	2.2
1	A	423	THR	2.1

Continued on next page...

CONFIDENTIAL VALIDATION REPORT

Continued from previous page...

Mol	Chain	Res	Type	RSRZ
1	A	424	PRO	2.1
1	A	255	ASN	2.1
1	A	449	GLU	2.1
1	A	104	LYS	2.1
1	A	331	TRP	2.1
1	A	103	PHE	2.1
1	A	59	VAL	2.1
1	A	141	GLN	2.0
1	A	453	THR	2.0
1	A	446	ALA	2.0

6.2 Non-standard residues in protein, DNA, RNA chains ⓘ

There are no non-standard protein/DNA/RNA residues in this entry.

6.3 Carbohydrates ⓘ

In the following table, the Atoms column lists the number of modelled atoms in the group and the number defined in the chemical component dictionary. The B-factors column lists the minimum, median, 95th percentile and maximum values of B factors of atoms in the group. The column labelled 'Q< 0.9' lists the number of atoms with occupancy less than 0.9.

Mol	Type	Chain	Res	Atoms	RSCC	RSR	B-factors($Å^2$)	Q<0.9
2	BMA	B	4	11/12	0.50	0.57	173,177,183,187	0
2	BMA	B	3	11/12	0.55	0.31	142,146,156,168	0
2	MAN	B	5	11/12	0.61	0.52	184,190,194,194	0
2	NAG	B	2	14/15	0.70	0.29	93,107,120,132	0
2	NAG	B	1	14/15	0.82	0.19	64,79,90,99	0

The following is a graphical depiction of the model fit to experimental electron density for oligosaccharide. Each fit is shown from different orientation to approximate a three-dimensional view.

CONFIDENTIAL VALIDATION REPORT

Electron density around Chain B:

$2mF_o$-DF_c (at 0.7 rmsd) in gray
mF_o-DF_c (at 3 rmsd) in purple (negative)
and green (positive)

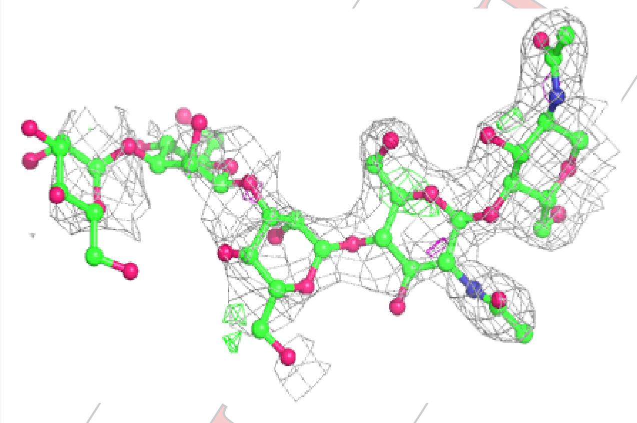

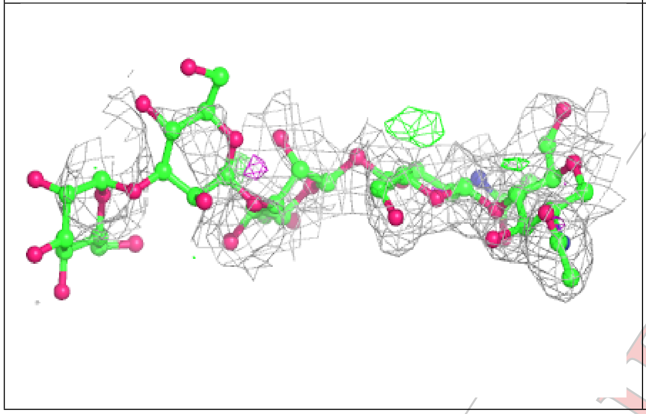

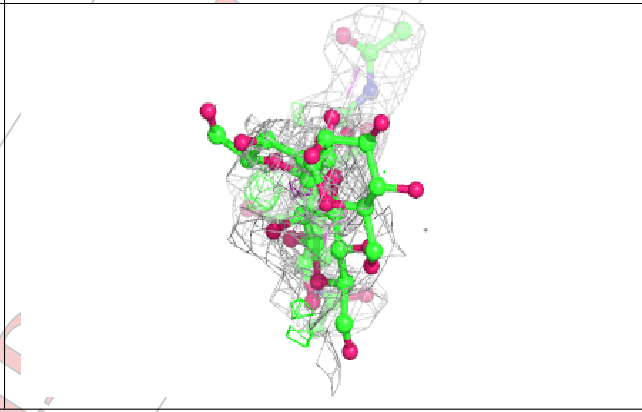

6.4 Ligands (i)

In the following table, the Atoms column lists the number of modelled atoms in the group and the number defined in the chemical component dictionary. The B-factors column lists the minimum, median, 95th percentile and maximum values of B factors of atoms in the group. The column labelled 'Q< 0.9' lists the number of atoms with occupancy less than 0.9.

Mol	Type	Chain	Res	Atoms	RSCC	RSR	B-factors(Å^2)	Q<0.9
3	NAG	A	601	14/15	0.51	0.66	141,156,162,164	0
4	GOL	A	604	6/6	0.73	0.36	148,153,154,154	0
5	PEG	A	606	7/7	0.80	0.24	92,93,109,110	0
4	GOL	A	602	6/6	0.84	0.31	94,99,105,108	0
5	PEG	A	605	7/7	0.88	0.28	79,89,117,117	0
4	GOL	A	603	6/6	0.92	0.24	93,98,100,104	0

6.5 Other polymers (i)

There are no such residues in this entry.
