# Supplementary figures and images for: Monomeric prefusion structure of an extremophile gamete fusogen and stepwise formation of the postfusion trimeric state
Source: Nat Commun. 2022 Jul 13;13:4064. doi: 10.1038/s41467-022-31744-z (PMC9279424; doi:10.1038/s41467-022-31744-z)

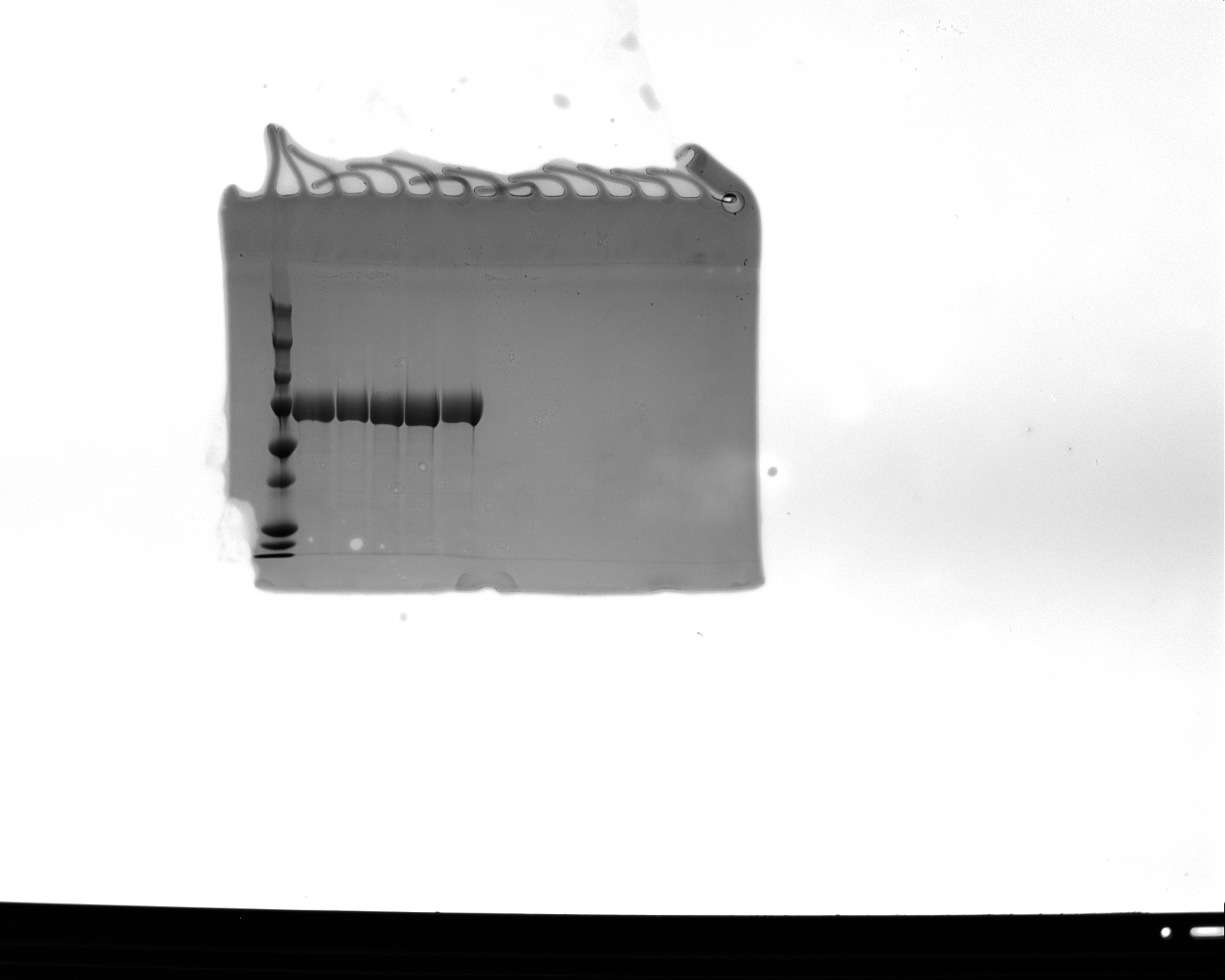

Supplement: Supplementary file 4 — Source Data [file 41467_2022_31744_MOESM4_ESM.zip › NC_Source-Data_NCOMMS-21-43412B/Figure8B_SDS-PAGE.jpg]
